# Associations of American Indian Children’s Screen-Time Behavior With Parental Television Behavior, Parental Perceptions of Children’s Screen Time, and Media-Related Resources in the Home

**Published:** 2011-08-15

**Authors:** Daheia J. Barr-Anderson, Jayne A. Fulkerson, Mary Smyth, John H. Himes, Peter J. Hannan, Bonnie Holy Rock, Mary Story

**Affiliations:** University of Minnesota, School of Kinesiology; University of Minnesota, Minneapolis, Minnesota; University of Minnesota, Minneapolis, Minnesota; University of Minnesota, Minneapolis, Minnesota; University of Minnesota, Minneapolis, Minnesota; University of Minnesota, Minneapolis, Minnesota; University of Minnesota, Minneapolis, Minnesota

## Abstract

**Introduction:**

American Indian children have high rates of overweight and obesity, which may be partially attributable to screen-time behavior. Young children's screen-time behavior is strongly influenced by their environment and their parents' behavior. We explored whether parental television watching time, parental perceptions of children's screen time, and media-related resources in the home are related to screen time (ie, television, DVD/video, video game, and computer use) among Oglala Lakota youth residing on or near the Pine Ridge Reservation in South Dakota.

**Methods:**

We collected baseline data from 431 child and parent/caregiver pairs who participated in Bright Start, a group-randomized, controlled, school-based obesity prevention trial to reduce excess weight gain. Controlling for demographic characteristics, we used linear regression analysis to assess associations between children's screen time and parental television watching time, parental perceptions of children's screen time, and availability of media-related household resources.

**Results:**

The most parsimonious model for explaining child screen time included the children's sex, parental body mass index, parental television watching time, how often the child watched television after school or in the evening, parental perception that the child spent too much time playing video games, how often the parent limited the child's television time, and the presence of a VCR/DVD player or video game player in the home (F_7,367_ = 14.67; *P* < .001; adjusted *R* ^2^ = .37). The presence of a television in the bedroom did not contribute significantly to the model.

**Conclusion:**

Changes in parental television watching time, parental influence over children's screen-time behavior, and availability of media-related resources in the home could decrease screen time and may be used as a strategy for reducing overweight and obesity in American Indian children.

## Introduction

Prevalence rates of overweight and obesity are high among all Americans (1), disproportionately so among American Indians (2-4). Data from the Early Childhood Longitudinal Study indicate that almost one-third of American Indian 4-year-old children are obese (body mass index [BMI] ≥95th percentile for same-age children), compared with 22.0% for Hispanic, 20.8% for non-Hispanic black, 15.9% for non-Hispanic white, and 12.8% for Asian children (5).

Childhood overweight and obesity are associated with a complex interplay of factors related to physical activity, screen time behavior, and diet (6). Behavior of children, especially those of preschool age, is strongly influenced by their environment and their parents' behavior (7). Although there is a body of research about the parental role in children's physical activity and dietary behaviors (7), few studies have examined the roles that parental behavior and perceptions and home environment may have on young children's screen-time behavior. Research on correlates of sedentary behavior has examined mainly psychosocial constructs rather than environmental factors and has focused on adolescent populations (8).

Given the current recommendations to begin obesity prevention efforts with preschoolers (9), understanding parental roles regarding obesity-influencing social (ie, parental behavior and perceptions) and physical (ie, media-related resources) factors in the home environment and how they relate to children's screen time behavior may inform strategies to reduce this sedentary behavior. The objective of this study was to examine the associations of children's screen time (ie, television, DVD/video, video game, and computer use) with parental television watching time, parental perceptions of children's screen time, and media-related resources (eg, number of televisions) in the home, among Oglala Lakota youth residing on or near the Pine Ridge Reservation in South Dakota.

## Methods

This study is a secondary data analysis of baseline data from an obesity prevention intervention. We approached school administrators and school boards of the 14 elementary schools located on or near the Pine Ridge Reservation in South Dakota. Each school agreed to participate in Bright Start, a 2-year, group-randomized, controlled trial, from 2006 to 2008. The study targeted American Indian students enrolled in kindergarten from all of the elementary schools located on or near the reservation. The focus of Bright Start was to create dietary and physical activity environmental changes at school and in the home to increase physical activity and healthy dietary practices to reduce excessive weight gain among American Indian youth.

We held Bright Start information meetings at each elementary school's fall open house. During these face-to-face meetings, we described all aspects of the study to the parents or caregivers (hereafter referred to as parents) and encouraged them to take a packet of information home to review. If the parents were interested, they returned the signed consent letter in a sealed Bright Start envelope to their child's teacher. For parents who did not attend the open house events, we sent the packets of information home with the child. The cover letter asked parents to carefully review the information and if they were interested in participating in the study to return the signed consent letter in a sealed Bright Start envelope to their child's teacher. Participation in all measurements was voluntary. Of the 472 total kindergarten students in the 14 schools at baseline, 454 (96%) were measured for height and weight; 431 parents (95%) completed a written survey. We received ethical approval for this study from the institutional review boards (IRBs) of the University of Minnesota, the Oglala Sioux Tribal Council, and the Aberdeen Area Indian Health Service. The Oglala Sioux and Aberdeen Indian Health Service IRBs also approved this manuscript before journal submission.

### Data collection

Parents completed a 2-part written survey; the first part asked about their child's behavior and the second part asked about their own behavior. Only baseline data from this survey were used in the current study.

Parents reported their child's and their own date of birth and sex. We calculated age at time of survey from the date of birth. Using standard protocols (10), we collected height in meters and weight in kilograms for both children and parents. BMI was calculated as kilograms divided by height in meters squared, and weight status categories were created for both children and parents. For children, Centers for Disease Control and Prevention BMI percentiles for age and sex were used to define underweight as BMI less than 5th percentile, normal weight as 5th to 85th percentile, overweight as 85th to less than 95th percentile, and obese as 95th percentile or higher. For adults, normal weight was defined as BMI less than 25.0 kg/m^2^, overweight as 25.0 to 29.9 kg/m^2^, and obese as 30.0 kg/m^2^ or higher. Because poverty is widespread on the reservation (11), a relative socioeconomic status variable was created by using factor analysis. This variable is a composite of 5 areas relevant to socioeconomic status (educational levels of parents, household income, unemployment status, public assistance benefits, resources in the home) and is applicable to this American Indian population only.

The questions used to assess children's screen time, parental television watching time, and parental perceptions of children's screen-time behavior were based on previous research (12). The variables examined in the current study were the child's daily screen time, parental daily television behavior, parental perception of the child's screen time, parental perception of the child's television behavior, inventory of media-related household resources (television, computer, Internet access, VCR/DVD player, video game player, and cable or satellite), and presence of a television in the child's bedroom (Appendix).

### Data analysis

We calculated frequencies, means, and standard deviations (SDs) to describe child and parental demographic characteristics, including age, sex, BMI, and BMI *z* score (children only). We conducted a series of stepwise linear regression analyses to assess the associations between 5 blocks of variables (parental television watching time, parental perceptions of the child's screen time, parental perceptions of the child's television behavior, media-related household resources, and presence of television in the child's bedroom) with the main outcome of children's screen time. Seven models were tested to derive the most parsimonious model that explained the most variance of children's screen time. The full model included all variables; subsequent submodels eliminated blocks of psychosocial and resource variables that were theoretically associated with the outcome; and the final model included only variables that were consistently significant throughout the submodels. Intervention condition, child's sex, and parental BMI were retained in each model as covariates because they each differed by child's screen time in preliminary analyses. Child's BMI was not included in the regression model because it was not associated with the outcome variable. All models were adjusted for relative socioeconomic status. Adjusted *R* ^2^ values were assessed for each model to determine parsimony. The dependent variable, children's screen time, was skewed and was log-transformed for analysis. For ease of interpretation, we present regression coefficients from the untransformed analysis and the *P* values from the transformed analysis. We completed all analyses using SAS version 9.2 (SAS Institute, Inc, Cary, North Carolina).

## Results

### Descriptive characteristics

The mean age of children in this sample was 5.8 years (SD, 0.51 y), and 30% of them were overweight or obese. Approximately 90% of the parents who completed the survey were female, and their mean BMI was 32.5 (SD, 7.34) kg/m^2^ (Table 1). Parents reported that their child engaged in 3.0 hours of screen time per day, while they themselves engaged in slightly less (2.4 h/d) (Table 2). Approximately 23% of parents strongly agreed or agreed that their child spent too much time watching television, and approximately 81% felt that it would be easy for them to limit their child's television time. Watching television after school (12.9%) was more than twice as common as watching television before school (4.5%). Presence of media-related items ranged from 31% who had Internet access to 93% who had a VCR/DVD player. Only 5 households did not own a television; more than two-fifths owned 3 or more televisions. More than half of the children had a television in their bedroom (Table 2).

### Regression analyses for child's screen time

The regression full model and submodel 5 explained the most variance of children's screen time; however, the final model was the most parsimonious (F_7,367_ = 14.67; *P* < .001), accounting for 37% of the variance with only 8 independent variables (Table 3). Variables that were significantly and positively associated with children's screen time were the child's sex (male), parental television watching time, parental perception that the child spent too much time playing video games, how often the child watched television after school or in the evening, and presence of a VCR/DVD player or a video game player in the home. Parental BMI and parental perception of how often they limit their child's television time were significantly and inversely associated with children's screen time.

More specifically, according to parental report, boys engaged in 0.30 more hours of screen time per day than girls. Children's screen time decreased by 0.02 hours per day for every 1 kg/m^2^ increase in parental BMI. Children's screen time increased by 0.37 hours per day for every 1-hour increase in parental television watching time. Children whose parents agreed with the statement that they spent too much time playing video games engaged in 1.06 more hours of screen time per day than children whose parents disagreed with this statement. Children whose parents responded that they often or always watched television after school or in the evening engaged in 1.0 more hours of screen time per day than children whose parents responded *never*, *rarely*, or *sometimes* to this statement. Children whose parents often or always limited child television time engaged in .38 fewer hours of screen time per day than children whose parents did not limit television time. Parents of children who had a working VCR/DVD player or video game player reported that their child engaged in 0.70 and 0.33 more hours of screen time, respectively, than parents of children who did not have those resources in the home.

## Discussion

Children's screen-time was associated with child's sex, parental BMI and television watching time, parental perceptions that the child spent too much time playing video games, how often the child watched television after school or in the evening, how often parents limited their child's television time, and presence of a VCR/DVD player or a video game player in the home. Television and media have a consistent presence in children's lives (13), and this may not change in the near future. Especially at this young age, parental influence (ie, behavior, perceptions, and availability of household resources) is pivotal in determining children's behavior, as our study demonstrates. The amount of time parents reported that their children engaged in screen time (3.0 h/d) is slightly lower than what has been reported in other studies for similar age groups. For example, parents of 5- to 6-year-olds reported almost 4 hours of screen time (watching television or DVDs, playing video games, or using the computer) on the previous day (13). Less screen time was reported among girls and children whose parents limited their child's television watching. Similar findings related to sex were reported in a sample of non–American Indian 5th- and 6th-graders (14) and similar findings of parental rules related to television use were reported in a sample of non–American Indian 11- to 15-year-olds (15).

The unexpected negatively associated factor in the current study was parental BMI; child's screen time was lower by 0.02 hours per day for every 1-unit increase in parental BMI. The relationship between television behavior and weight status was moderated by parental weight in samples of older children and adolescents (ie, 10- to 13-year-old girls and 14- to 19-year-old boys) but not for children aged 6 to 9 years (16). A cross-sectional study of 8-, 11-, and 14-year-olds found that children and adolescents who have at least 1 overweight parent watched more hours of television than those with 2 normal-weight parents (17). It is not clear why the relationship between parental BMI and child screen time is more apparent in older children and adolescents, but it could be because data about younger children's behavior (<9 y) are reported by the parent rather than self-reported, which is the usual method for older children and adolescents. Because of social desirability, parents of younger children may be underestimating their child's screen time behavior, which could bias results.

As expected, parental television watching time and parental perception that the child spent too much time playing video games were associated with increased children's screen time. Parental role modeling of sedentary behavior has consistently been associated with increased sedentary behavior in their children (18,19).

It may be assumed that the availability of media-related resources in the home would increase the time spent engaged in screen time. However, of the 7 resources that were examined in the current study (household television, computer, Internet access, DVD/VCR player, video game player, cable or satellite, and bedroom television) only the availability of a VCR/DVD player or a video game player was associated with increased screen time in young American Indian children. On a national level, which is also reflected in the current study, more than 90% of parents of children aged 5 to 6 years report having a VCR/DVD player in the home (13). It is plausible that the relationship seen between the availability of a VCR/DVD player and children's screen time may be exacerbated because of the oversaturation of this piece of media-related equipment in households.

The increased use of sedentary video games has been cited as a possible contributing factor to obesity in children. About two-thirds of the current sample reported owning a video game player; the national average for 5- to 6-year-olds is 52% (13). Younger children who played a video game during the previous day tended to spend more than 50 minutes playing electronic games (13), which parallels the findings from this study.

The presence of a bedroom television is associated with increased screen time in older adolescents aged 15 to 18 years (20). However, this relationship is not evident in young children in a nationally representative sample of 8- to 18-year-olds (21) or consistently present in our study. The American Academy of Pediatrics recommends that parents restrict their children from having a television in their bedrooms (22), but 51% of the current study's sample and 37% of 5- to 6-year-olds nationally have a bedroom television (13). However, neither the current study nor a study based on a representative sample (13) supported the hypothesis that the presence of a television in the child's bedroom was associated with children's screen time. Although young children may have access to a television, the television may not have as much of an influence on their viewing because younger children may not have the same level of autonomy as older children and adolescents to choose when and what they watch on television. Data are unavailable for preschool-aged children, but nationally representative data support that 66% of 8- to 10-year-olds compared with 51% of 11- to 14-year-olds and 26% of 15- to 18-year-olds reported their parents have rules about which shows they can watch on television (21). It is reasonable to conclude that even more than 66% of 5- to 6-year-olds would have such restrictions.

Major strengths of this study are that findings add to the sparse literature on American Indian children and obesity, the population of focus was younger children rather than adolescents, and both physical (ie, media-related resources) and social (ie, parental perceptions) factors were examined. One study limitation is the potential bias for social desirability since data were collected only via parent report. The cross-sectional design of the study prohibits cause-effect inferences; however, our study is a necessary, initial step when exploring mechanisms that may lead to changes in behavior (23). Although our findings may contribute to the prevention of overweight and obesity by limiting sedentary behavior, they are limited to preschool-aged American Indian children. Given that this population is at considerable risk for obesity, these findings may inform the types of attitudes and behaviors that can be targeted for obesity prevention and intervention.

Positive parental involvement and role modeling are necessary for reducing and preventing overweight and obesity at a young age. Parents need to lead by example and teach their children healthy lifestyle behaviors. Results from the current study suggest that changes in modifiable factors such as parental television watching time, parental perceptions of child screen time, and the availability of media-related resources merit further investigation as a means to decrease screen time and consequent overweight and obesity in American Indian children.

## Figures and Tables

**Table 1:** Demographic Characteristics of American Indian Children and Their Parents

**Characteristic**	n^a^	% or Mean (SD)
**Children**		
**Age, mean (SD), y**	431	5.8 (0.51)
**Sex, %**		
Female	211	49.0
Male	220	51.0
**BMI, mean (SD), kg/m^2^**	423	16.6 (2.87)
**BMI, mean (SD), *z* score**	423	0.49 (1.17)
**Weight status, %^b^**		
Underweight	19	4.5
Normal weight	279	66.0
Overweight	61	14.4
Obese	64	15.1
**Parents**		
**Age, mean (SD), y**	427	35.7 (1.73)
**Sex, %**		
Female	385	89.3
Male	46	1.7
**BMI, mean (SD), kg/m^2^**	430	32.5 (7.34)
**Weight status, %^c^**		
Normal weight	61	14.2
Overweight	105	24.4
Obese	264	61.4

Abbreviations: SD, standard deviation; BMI, body mass index.

a Sample sizes may vary because of incidental missing values.

b Based on Centers for Disease Control and Prevention BMI percentiles for age and sex. Normal weight defined as ≥5th but <85th percentile; overweight defined as ≥85th but <95th percentile; obese defined as ≥95th percentile.

c Normal weight defined as BMI <25.0 kg/m^2^; overweight defined as BMI 25.0-29.9 kg/m^2^; obese defined as BMI ≥30.0 kg/m^2^.

**Table 2:** Characteristics of American Indian Children's Screen Time Behavior, Parental Television Watching Time, Parental Perceptions of Children's Screen Time Behavior, and Media-Related Household Resources

**Screen Time/Behavior**	n^a^	Mean (SD)
Child's screen time, h/d^b^	423	3.0 (1.84)
Parental television watching time, h/d	423	2.4 (1.85)

**Perceptions/Resources**	n^a^	%

**Parental perception of child's screen time, % strongly agree or agree^c^**		
Too much time watching television	420	22.6
Too much time playing video games	417	15.1
Easy to limit child's television	421	81.2
**Parental perception of child's television behavior, % often or always^d^**		
Watch television before school	420	12.9
Watch television after school/evenings	420	4.5
Television on when not watching	420	42.6
How often limit child's television	420	44.3
**Media-related household resources, % yes**		
≥3 Televisions	408	42.7
Computer	421	43.0
Internet access	417	31.2
VCR/DVD player	422	93.4
Video game player	421	65.8
Cable or satellite	419	67.1
**Television in child's bedroom, % yes**	421	51.3

Abbreviation: SD, standard deviation.

a Sample sizes may vary slightly because of incidental missing values.

b Screen time includes time spent watching television or videos and time spent playing video games or using the computer.

c These 3 items do not constitute a scale.

d These 4 items do not constitute a scale.

**Table 3:** Associations of American Indian Children's Screen-Time Behavior With Parental Television Watching Time, Parental Perceptions of Children's Screen Time, and Media-Related Resources in the Home^a^

Characteristic	Full Model		Submodel 5		Final Model	

	β	*P* Value	β	*P* Value	β	*P* Value
**Condition (intervention vs control)**	.05	.73	.07	.68	.10	.50
**Child's sex**	.35	.03	.33	.04	.30	.04
**Parental BMI**	–.03	.01	–.03	.01	–.02	.01
**Parental television watching time (h/d)**	.35	<.001	.36	<.001	.37	<.001
**Parental perception of child's screen time (agree/disagree)**						
Too much time watching television	–.03	.90	–.04	.84	NI	NA
Too much time playing video games	.82	.001	.91	<.001	1.06	<.001
Easy to limit child's television	.02	.92	–.02	.91	NI	NA
**Parental perception of child's television behavior (often/always vs never/rarely/sometimes)**						
Watch television before school	.13	.60	.11	.65	NI	NA
Watch television after school/evenings	1.07	<.001	1.11	<.001	1.00	<.001
Television on when not watching	.08	.62	.10	.56	NI	NA
How often limit child's television	–.35	.03	–.38	.02	–.38	.01
**Media-related household resources (yes/no)**						
≥3 Televisions	–.27	.12	–.18	.28	NI	NA
Computer	–.29	.15	–.29	.15	NI	NA
Internet access	.10	.64	.11	.62	NI	NA
VCR/DVD player	.86	.01	.86	.01	.70	.02
Video game player	.42	.02	.42	.02	.33	.04
Cable or satellite	–.02	.90	–.01	.97	NI	NA
**Presence of television in child's bedroom (yes/no)**	.28	.10	NI	NA	NI	NA
**Adjusted *R* ^2^**	.38		.38		.37	

Abbreviations: BMI, body mass index; NI, variable not included in model; NA, not applicable.

a The full model included all variables; 5 subsequent submodels eliminated blocks of psychosocial and resource variables that were theoretically associated with the outcome, and the final model included only variables that were consistently significant throughout the submodels. All models retained intervention condition, child's sex, and parental BMI and were adjusted for relative socioeconomic status, a variable that is a composite of 5 areas relevant to socioeconomic status.

## Appendix: Questions and Response Categories for Children's Screen Time Behavior, Parental Television Watching Time, Parental Perceptions of Children's Screen Time, and Media-Related Household Resources

**Table:**

**Question**	Response Category
**Child's screen time behavior^a^^,^ b**	
About how many hours a day on school days does your child watch TV or videos? (Do not count hours at school.)	0, <1, 1-2, 3-4, 5-6, ≥7
About how many hours a day on weekend or non-school days does your child watch TV or videos?	
About how many hours a day on school days does your child play video games or use the computer? (Do not count hours at school.)	
About how many hours a day on weekend or non-school days does your child play video games or use the computer?	
**Parental television watching time^a^**	
About how many hours a day do you watch TV or videos?	0, <1, 1-2, 3-4, 5-6, ≥7
**Parental perception of child's screen time**	
How much do you agree or disagree with the following statements about your child's activity:	
My child spends too much time watching TV.	Strongly agree, agree, disagree, strongly disagree
My child spends too much time playing video games.	
It is usually easy for me to limit the amount of TV my child watches.	
**Parental perception of child's television behavior**	
How often does your child watch TV before school?	Never, rarely, sometimes, often, always
How often does your child watch TV after school or evening?	
How often is a TV on in your house even when no one is watching it?	
How often do you put limits on how much time your child may watch TV?	
**Media-related household resources**	
Do you have any of the following items in your household? Count only those items that are in working condition.	
Television	Yes, no
If yes, how many?	1, 2, ≥3
Computer	Yes, no
Internet access	Yes, no
VCR/DVD player	Yes, no
Video game player	Yes, no
Cable or satellite	Yes, no
**TV in child's bedroom**	
Does your child have a TV in his or her bedroom?	Yes, no

a The responses for each of the 4 questions (0, <1,1-2, 3-4, 5-6, or ≥7h/d) were recoded (0, .5, 1.5, 3.5, 5.5, or 7 h/d) and summed to create a score that represented the total time spent engaged in sreen time.

b Recoded responses from these 4 items were summed and weighted to create daily average of screen-time use. Response categories were the same for all 4 items.

## References

[1] Flegal KM, Carroll MD, Ogden CL, Curtin LR (2010). Prevalence and trends in obesity among US adults, 1999-2008. JAMA.

[2] Broussard BA, Johnson A, Himes JH, Story M, Fichtner R, Hauck F (1991). Prevalence of obesity in American Indians and Alaska Natives. Am J Clin Nutr.

[3] Broussard BA, Sugarman JR, Bachman-Carter K, Booth K, Stephenson L, Strauss K, Gohdes D (1995). Toward comprehensive obesity prevention programs in Native American communities. Obes Res.

[4] Welty TK, Lee ET, Yeh J, Cowan LD, Go O, Fabsitz RR (1995). Cardiovascular disease risk factors among American Indians. The Strong Heart Study. Am J Epidemiol.

[5] Anderson SE, Whitaker RC (2009). Prevalence of obesity among US preschool children in different racial and ethnic groups. Arch Pediatr Adolesc Med.

[6] Anderson PM, Butcher KE (2006). Childhood obesity: trends and potential causes. Future Child.

[7] Olstad DL, McCargar L (2009). Prevention of overweight and obesity in children under the age of 6 years. Appl Physiol Nutr Metab.

[8] Van Der Horst K, Paw MJ, Twisk JW, Van Mechelen W (2007). A brief review on correlates of physical activity and sedentariness in youth. Med Sci Sports Exerc.

[9] Koplan JP, Liverman CT, Kraak VI (2005). Preventing childhood obesity: health in the balance.

[10] Lohman TG, Roche AF, Martorell R (1988). Human Kinetics.

[11] Indian Health Service (1996). US Department of Health and Human Services.

[12] Story M, Sherwood NE, Himes JH, Davis M, Jacobs DR, Cartwright Y (2003). An after-school obesity prevention program for African American girls: the Minnesota GEMS pilot study. Ethn Dis.

[13] Vandewater EA, Rideout VJ, Wartella EA, Huang X, Lee JH, Shim MS (2007). Digital childhood: electronic media and technology use among infants, toddlers, and preschoolers. Pediatrics.

[14] He M, Harris S, Piché L, Beynon C (2009). Understanding screen-related sedentary behavior and its contributing factors among school-aged children: a social-ecologic exploration. Am J Health Promot.

[15] Norman GJ, Schmid BA, Sallis JF, Calfas KJ, Patrick K (2005). Psychosocial and environmental correlates of adolescent sedentary behaviors. Pediatrics.

[16] Vandewater EA, Huang X (2006). Parental weight status as a moderator of the relationship between television viewing and childhood overweight. Arch Pediatr Adolesc Med.

[17] Steffen LM, Dai S, Fulton JE, Labarthe DR (2009). Overweight in children and adolescents associated with TV viewing and parental weight: Project HeartBeat!. Am J Prev Med.

[18] Jago R, Fox KR, Page AS, Brockman R, Thompson JL (2010). Parent and child physical activity and sedentary time: do active parents foster active children?. BMC Public Health.

[19] Jago R, Page A, Froberg K, Sardinha LB, Klasson-Heggebo L, Andersen LB (2008). Screen-viewing and the home TV environment: the European Youth Heart Study. Prev Med.

[20] Barr-Anderson DJ, van den Berg P, Neumark-Sztainer D, Story M (2008). Characteristics associated with older adolescents who have a television in their bedrooms. Pediatrics.

[21] Rideout VJ, Foehr UG, Roberts DF (2001). Generation M2: media in the lives of 8- to 18-year olds.

[22] American Academy of Pediatrics Committee on Public Education (2001). American Academy of Pediatrics: children, adolescents, and television. Pediatrics.

[23] Bauman AE, Sallis JF, Dzewaltowski DA, Owen N (2002). Toward a better understanding of the influences on physical activity: the role of determinants, correlates, causal variables, mediators, moderators, and confounders. Am J Prev Med.

